# Gender Socialization as a Predictor of Psychosocial Well-Being in Young Women with Breast Cancer

**DOI:** 10.3390/curroncol29110641

**Published:** 2022-10-27

**Authors:** Lianne Trachtenberg, Mary Jane Esplen, Brenda Toner, Niva Piran

**Affiliations:** 1 Navigate Clinic, Brookdale Ave, Toronto, ON M5M 1P8, Canada; 2 Department of Psychiatry, Temerty Faculty of Medicine, University of Toronto, 250 College Street, Room 830, Toronto, ON M5T 1R8, Canada; 3 Department of Applied Psychology and Human Development, OISE, University of Toronto, 252 Bloor Street West, Toronto, ON M5S 1V6, Canada

**Keywords:** breast cancer, gender socialization, young women, psychosocial well-being, cancer survivorship

## Abstract

This study aimed to explore the relationship between gender socialization and psychosocial well-being among young women diagnosed with and treated for breast cancer. A total of 113 women between the ages of 18–49 completed a one-time questionnaire package. Four key measures of gender socialization were included: Gender Role Socialization Scale (GRSS), Objectified Body Consciousness Scale (OBCS), Mental Freedom Scale (MFS), and Silencing the Self Scale (SSS). Two measures of psychosocial well-being were included: Functional Assessment of Cancer Therapy-Breast (FACT-B) and Experience of Embodiment Scale (EES). Correlational and regression analyses were conducted to assess the relationship between gender socialization variables and well-being. In multiple regression models, GRSS and MFS added significant increments to the prediction of variance of the FACT-B (*R*^2^ = 23.0%). In contrast, the OBCS and MFS added significant increments to the prediction of variance of the EES (*R*^2^ = 47.0%). Findings suggested that women with greater endorsements to proscribed gender socialization were associated with poor well-being scores. Women who endorsed a critical stance, resisting traditional gender-role expectations, objectification pressures, and other social discourses, were associated with greater well-being scores. Future studies are needed to examine the impact of gender socialization on the well-being of young people with breast cancer.

## 1. Introduction

Breast cancer in young women is likely to be fast-growing and high-grade [1,2,3]. As a result, treatment can include a combination of surgery, chemotherapy, radiation, and hormone therapy. The multimodal method of treatment can improve long-term survival outcomes but can also contribute to prolonged periods of medical intervention with associated physical and emotional sequelae [4]. Young women are increasingly more vulnerable to poor psychosocial adjustment [4,5,6]. Past research has assessed a diverse range of factors associated with poor psychosocial well-being among young women with breast cancer, including body-image distress, sexual dysfunction, infertility, unemployment, and fears of recurrence [7,8,9,10]. One variable that has received little attention is gender socialization. Gender socialization is “the process whereby individuals develop, refine, and learn to ‘do’ gender through internalizing gender norms and roles as they interact with key agents of socialization, such as their family, social networks, and other social institutions” [11] (p. 15). Young women with breast cancer, whose bodies, including their breasts, hair, vaginal tissue, fertility status, energy levels, and weight, are affected, can be challenged to meet idealized heteronormative standards of femininity and appearance. Four unique components of gender socialization have previously been discussed as contributing to women’s overall psychosocial well-being after diagnosis and treatment of breast cancer [12,13].

The first component believed to influence well-being is gender-role socialization. Gender-role socialization involves a woman’s internalization of and compliance with socially accepted standards of role demands and physical appearance [13]. Direct and indirect communications from various influential sources (media, family, and peers) reinforce cultural heteronormative ideals that women are encouraged to adopt in order to gain societal approval, such as “the selfless caretaker”, “the nurturing child-bearer”, and/or “the sexual lover” [14,15]. However, research has shown that it is not the continuous bombardment of media messages that causes distress per se but the extent to which an individual internalizes and integrates these messages, thereby contributing to their self-concept [15]. For example, women who endorse traditional gender-role expectations and attitudes have a tendency to internalize cultural beauty standards and hold greater investment in their physical appearance [16]. Not surprisingly, research has demonstrated that women with breast cancer who held greater investment in physical appearance exhibited increased difficulty adjusting post-treatment relative to those with low levels of investment [17,18].

The second component associated with gender socialization and believed to influence well-being is self-objectification. According to objectification theories [19,20], self-objectification is defined as the internalization of the outsider gaze on the physical self. Calogero and colleagues [21] explained that “women learn to restrict their physical and social movement, investing their energy and resources in anticipation of the evaluative sexualized gaze” (p. 8). Higher self-objectification has been associated with disordered eating, depression, sexual dysfunction, and greater body shame in Western cultures [19]. More recently, research has begun establishing the link between self-objectification and body-image disturbances in women’s level of psychosocial well-being following breast cancer treatment [5,15,22].

A third, related component of gender socialization and overall well-being is mental freedom. According to Piran’s [23] Developmental Theory of Embodiment, mental freedom relates to “holding a critical stance toward, and experiencing freedom from, constraining social discourses that regulate embodied lives of individuals of different social locations” (p. 111). As individuals comply with constraining socially created molds, their possibilities of engaging meaningfully and passionately with the world become limited, restricting agency and self-attunement. Socially created molds are sometimes expressed through regulating labels [23,24]. For young women with breast cancer, whose bodies have been altered (e.g., breasts, hair, vaginal tissue, weight), a critical stance against idealized gendered labels (e.g., feminine, sexy, nurturing), as well as new negative illness-related labels (e.g., “damaged goods”, patient, sick), can occur [5].

The fourth component related to gender socialization and women’s overall well-being is silencing the self. This construct relates to stereotyped social expectations that women may silence their own needs and feelings of anger in relationships while also caring for others in order to maintain relational connections [25,26]. Silencing the self is based on a model suggesting that cognitive schemas target the creation and maintenance of safe and intimate relationships, leading women to silence certain thoughts, feelings, and actions. Among young women with breast cancer, the illness and its associated treatments can result in some feeling unable to share their personal thoughts, feelings, or behaviors in order to avoid burdening loved ones; women can also experience shame associated with their altered bodies, resulting in greater silencing of the self and stressors in their relationships.

Within psycho-oncology, few quantitative inquiries have explored gender socialization within the context of well-being and body-image distress among breast cancer patients. In one instrumental study, Boquiren and colleagues [15] examined the relationship between gender-role socialization and body objectification in body-image disturbances among 150 breast cancer survivors, ages 26–75 (*M* = 49.5, *SD* = 8.8). They found significant positive correlations between body-image distress and (a) gender-role socialization and (b) self-objectification measures. Further, body-image distress was negatively associated with quality of life (QOL). A path analysis was also conducted, delineating pathways between those psychosocial variables and body-image distress. These findings suggested that women with breast cancer who endorsed greater internalizations of traditional feminine roles also engaged in self-surveillance and experienced body dissatisfaction and poor QOL.

The purpose of the current study was to expand upon the existing literature by examining four key yet distinct components of gender socialization: namely, gender-role socialization, self-objectification, mental freedom, and silencing of the self. Moreover, the current study specifically examined those four components in relation to the well-being of young women who have been diagnosed and treated for breast cancer. The hypotheses were as follows:

(1) Poor well-being scores will be associated with (a) greater endorsement of proscribed gender socialization as measured by the Gender Role Socialization Scale (GRSS), (b) greater self-objectifying behaviours as measured by the Objectified Body Consciousness Scale (OBCS), and (c) greater levels of self-silencing (SSS) as a reflection of internalized gendered scripts.

(2) Greater well-being scores will be associated with an increased critical stance toward oppressive social discourses, expectations, and labels as measured by the Mental Freedom Scale (MFS).

## 2. Materials and Methods

### 2.1. Participants

The study was coordinated at the Princess Margaret Cancer Centre (PMCC) and the University of Toronto in Toronto, Canada. Approval was obtained from the ethics committees of both participating institutions (protocol code: 30548). Women were recruited from Canadian cancer community organizations and from the PMCC. Community recruitment involved online advertisements from cancer organizations. In-person, hospital-based recruitment occurred at patient follow-up appointments. Participants were eligible if they (a) were diagnosed with breast cancer, (b) were between the ages of 18–49, (c) had completed primary cancer treatment(s) including mastectomy (with or without reconstruction), and (d) were proficient in speaking and reading English. Participants were deemed ineligible if their diagnosis was established above age 49. Women who provided informed consent were provided with a link to an online questionnaire package.

A total of 227 potential participants who had completed primary breast cancer treatment(s) were screened for eligibility. A total of 86 women did not meet inclusion criteria and were not eligible to begin the one-time questionnaire package. A group of 141 women met inclusion criteria and completed the informed consented process. A total of 113 women completed the full questionnaire package and were included in the analysis.

### 2.2. Measures

#### 2.2.1. Gender Socialization Measures

The Gender Role Socialization Scale (GRSS) is a standardized 30-item self-report measure that assesses the degree to which a woman internalizes gender-role norms prescribed by modern day society. This measure widens the scope of clinical focus from looking at individual pathology to possible restrictive and oppressive social structures that women inhabit as contributors to poor mental health and well-being [15]. Higher scores reflect a greater degree of internalization of gender-role norms. Examples of items include “If I don’t accomplish everything I should, then I must be a failure” and “Whenever I see media images of women, I feel dissatisfied with my body”. Toner and colleagues [27] as well as Boquiren and colleagues (2013) both found the scale to be reliable (Cronbach’s alpha = 0.93 and 0.88, respectively).

The Objectified Body Consciousness Scale (OBCS) is a standardized 24-item self-report scale designed to measure objectified body consciousness in women [15,28]. Women who report higher scores demonstrate greater objectified body consciousness. The OBCS contains three subscales: (a) Body Shame, which explores feelings of shame when the body fails to meet social norms—a typical item is “I feel like I must be a bad person when I don’t look as good as I could”; (b) Body Surveillance, which explores women who survey their body from an observer’s perspective—a typical item is “I rarely compare how I look with how other people look”; and (c) Appearance Control Beliefs, which measures beliefs about personal responsibility and control over one’s appearance—a typical item in this scale is “I think a person can look pretty much how they want to if they are willing to work at it”. Only the first two subscales, Body Shame and Body Surveillance, were used in the present study, as questions from the third subscale overlapped with items from the Mental Freedom scale. McKinley and Hyde [28] showed that the first two subscales, Body Shame and Body Surveillance, were two key factors that contributed to overall body dissatisfaction. Boquiren and colleagues (2013) found strong reliability in both the OBCS’ Body Shame and Body Surveillance subscales (Cronbach’s alphas = 0.77 and 0.81, respectively).

The Mental Freedom Scale (MFS) is a standardized 37-item self-report measure that assesses women’s critical stance toward oppressive social discourses, expectations, and labels [24]. Higher scores reflect women who were more resistant to oppressive social discourses. Items include “I have felt that being physically strong conflicts with being a girl/woman” and “I have been encouraged to think critically about different social pressures that I have experienced”. The items reflect collusion with or resistance and critical stance toward oppressive social discourses and expectations related to being a woman. This scale has a Cronbach’s alpha of 0.93 [29].

The Silencing the Self Scale (SSS) is composed of 31 items examining individuals’ beliefs and behaviours about interpersonal relationships [26,30]. The scale was derived from longitudinal data of clinically depressed women. High scores reflect a greater degree of self-silencing amongst women. Items include “Caring means putting the other person’s needs in front of my own” and “I don’t speak my feelings in an intimate relationship when I know they will cause disagreement” [30]. The scale has been found to have good internal consistency, with alpha coefficients ranging from 0.86 to 0.94 and test–retest reliability ranging from 0.88 to 0.93 [26]. The present study used the Silencing the Self subscale (SS-2) and Care as Self-Sacrifice subscale (SS-1), as they were believed to be most relevant to this study objectives.

#### 2.2.2. Measures of Psychosocial Well-Being

The Functional Assessment of Cancer Therapy—Breast (FACT-B) is a well-validated multi-dimensional self-report scale designed to assess QOL in women who have been diagnosed with breast cancer [31,32]. The total score on the FACT-B (Version 4) is composed of the FACT-General (FACT-G), which includes 27 items, plus 10 additional breast-cancer-related items. The FACT-G has four subscales, including (a) physical well-being, (b) social/family well-being, (c) emotional well-being, and (d) functional well-being. Higher scores are indicative of higher reported QOL levels.

The Experience of Embodiment Scale for Women (EES), developed by Piran and Teall [29], is a 34-item standardized multi-dimensional self-report scale designed to assess women’s embodied well-being: in particular, their “experience of engagement of the body with the world” [33] (p. 177). The EES includes an emphasis on women’s internal experiences, encompassing a broad range of experiences from embodied agency, positive connection, and self-care to restraint, disconnection, and harmful behaviours. The EES has five central dimensions: body connection and comfort, agency through physical activity and voice, experience and expression of desires, attuned self-care, and freedom from self-objectification [34]. Higher scores reflect greater experiences of embodied well-being. Examples of items include “I have cared more about how my body feels than about how it looks”, “I feel at one with my body”, and “my body has made me feel depressed/anxious”. The EES has a Cronbach’s alpha of 0.94 and was found to be significantly correlated with the Body Responsiveness Scale (*r* = 0.73) [35], the Body Esteem Scale (*r* = 0.78) [36], and the EAT-26 (*r* = −0.45) [37].

### 2.3. Data Analysis

Data analysis was conducted using SPSS Version 20. Multiple imputations were used to compute missing data; the iterative Markov Chain Monte Carlo method was used due to an arbitrary missing data pattern. An exploration of the original data set, five imputed data sets, and the pooled data set were explored to ensure there were no major discrepancies. Descriptive analyses were calculated for all study variables. Bivariate correlations were conducted to assess the relationships between dependent variables (FACT-B, EES) and independent variables (GRSS, OBCS, MFS, and SSS). A second set of independent sample t-tests and one-way ANOVAs were conducted to compare dependent variables to demographic and clinical variables. Any variable showing significance was included in the regression analyses as a covariate. Relationships between primary study variables were analyzed through correlational analyses.

## 3. Results

### 3.1. Participants

One hundred and thirteen participants completed the study; participants were between the ages of 18 and 49 (*M* = 36.25, *SD = 5.89*). The majority were of European origin (62.8%), had a college or undergraduate degree (56.7%), and worked full-time (55.8%). Most participants lived with a partner (69.9%) and had at least one dependent child (59.3%). The majority described being diagnosed with Stage III (31.9%) or Stage II (31.0%) cancer (See Table 1).

### 3.2. Descriptive Analysis

The descriptive analysis showed that the effects of occupation status (full time, part time, unemployed) on the FACT-B—F(2, 112) = 3.485, *p* = 0.031—and on the EES—F(2, 112) = 3.273, *p* = 0.038—were significant (see Table 2). A post-hoc multiple-comparisons test using Scheffé’s method found that the effects of the EES were significantly different for participants who reported being unemployed in relation to participants who reported being part-time/self-employed: *p* < 0.05. A second ANOVA showed that the stage of breast cancer (i.e., Stage 0-I, Stage II, Stage III-IV) on FACT-B was significant: F(2, 112) = 4.351, *p* < 0.013. The post-hoc multiple comparisons test using Scheffé’s method found that the effects of the FACT-B were significantly greater among participants with Stage II cancer diagnoses relative to participants with Stage 0-I cancer diagnoses: *p* < 0.05. In addition, significant positive correlations were found between FACT-B and current age—*r* = 0.20, *p* < 0.05—and EES and current age—*r* = 0.19, *p* < 0.05. No other significant associations were found between demographic variables and dependent variables. In light of these findings, occupational status, stage of breast cancer, and current age were included in step 1 of the regression analysis.

The relationship between clinical and gender socialization variables on well-being was analyzed through hierarchical regression analyses. Variables composing each block were entered into the analysis as follows: step 1—demographic and clinical variables (current age, full-time employment vs. unemployment, part-time employment vs. unemployment, Stage 0-I cancer vs. Stage III-IV cancers, Stage II cancer vs. Stage III and IV cancers); step 2—GRSS, OBCS, MFS, SSS. The *F change* ratio and adjusted *R^2^* were used to assess the fit of the regression models.

### 3.3. Relationship between Psychosocial Well-Being and Gender Socialization Measures

An initial set of correlational analyses were conducted to explore how both measures of well-being related to gender socialization measures, namely GRSS, OBCS, MFS, and SSS (see Table 3). Using the FACT-B and EES, women’s well-being was significantly associated with gender socialization variables. A greater degree of gender-role socialization, greater self-objectification, and a greater degree of self-silencing were negatively associated with women’s well-being following treatment(s). In addition, an increase in MFS (i.e., mental freedom—women’s critical stance toward oppressive social discourses, expectations, and gendered labels) was positively associated with well-being scores. The EES also demonstrated stronger correlations relative to the FACT-B among all independent variables. Correlation sizes among constructs were low to moderate (range = 0.22–0.45). All correlations were significant at the *p* < 0.01 level (see Table 3).

Two hierarchical regression analyses were conducted to test the contributions of the hypothesized study variables to women’s overall well-being. With regard to the FACT-B, the regression model identified a significant amount of variance: *F change* (6, 101) = 6.826, *p* < 0.001, *R*^2^*_change_* = 0.233. The main effect block was associated with another 23% of variance in FACT-B scores over and above the covariates (current age, occupation, and stage of cancer). Examination of independent variables within the main effect block showed that gender-role socialization (GRSS) and taking a critical stance toward oppressive social discourses (MFS) were significantly associated with the FACT-B (see Table 4).

With regard to the EES, the regression model was associated with a significant amount of variance above and beyond the covariates: *F change* (6, 101) *=* 22.853, *p* < 0.001, *R*^2^*_change_* = 0.467. The main effect block was associated with another 47% of variance in EES scores over and above current age, occupation, and stage of cancer. Examination of independent variables within the main effect block showed that the Objectified Body Consciousness Subscales—women’s experiences of body shame and surveillance—and women’s ability to take a critical stance toward oppressive social discourses (MFS) were significantly associated with the EES (see Table 5).

## 4. Discussion

The overall goal of the present study was to examine four key components of gender socialization and its association with well-being among young women diagnosed with and treated for breast cancer. Our findings lent empirical support to the hypothesized relationship between those constructs: that is, young women with breast cancer who resisted the internalization of traditional gender-role beliefs and formulated a critical stance toward gender-related social discourses, expectations, and labels were associated with high levels of social, psychological, spiritual, or physical functioning and embodied well-being following treatment.

The present study conducted two hierarchical regressions using the four gender socialization measures to examine their relationship to psychosocial well-being. The first regression, using a traditional medicalized measure of psychosocial well-being, found that gender-role socialization (GRSS) and mental freedom (MFS) added significant increments to the prediction of variance of the FACT-B. The second regression, using a nuanced measure specifically targeting women’s embodied well-being, demonstrated that women’s experiences of body shame, body surveillance, and mental freedom (MFS) added significant increments to the prediction of variance of the EES.

Consistent with previous findings, adjustments after completion of treatment may be challenging for women whose self-worth is directly linked to an adherence to traditional gender-role beliefs, as family and peers typically expected them to return to “normal” and resume routine home and work duties [15]. Among some women with breast cancer, who identified as having strong, traditional feminine identities such as “the selfless one”, “the emotional caretaker”, “the nurturer”, or “the sexual lover” prior to their diagnosis, those identities may no longer function and thus can negatively impact confidence and contribute to reduced well-being [15]. One possible explanation in regard to the associated relationship between gender-role socialization and the FACT-B, highlighted by Boquiren and colleagues [15], is that greater gender-role socialization exerts “a pressure on breast cancer survivors to continue to meet personal standards held prior to their illness” that is difficult if not impossible to attain.

The relationship between women’s body shame (i.e., feeling shame when the body does not conform to idealized representations of beauty), body surveillance (i.e., viewing the body as an outside observer would), and embodied well-being, as reflected by the EES, was in line with Fredrickson and Roberts’ [20] objectification theory. This theory suggested that objectification of women’s bodies, regardless of illness, leads to enhanced body shame and surveillance. Individuals with greater predisposition to self-objectify tended to show a chronic preoccupation with their physical appearance, with the belief that their bodies are and will be evaluated by others [19]. The experience of breast cancer can further promote body shame and surveillance due to a failure to comply with idealized representations of beauty; as a result, these factors may have played a critical role for the 20–45% of women with breast cancer who reported persistent forms of body-image distress after completing treatment [38]. For women with breast cancer, the comparison of physical appearance to internalized cultural expectations has been shown to contribute to body-image disturbances and negative self-views and interfere with adjustments to the task of rebuilding the post-cancer self.

Mental freedom, reflecting a critical stance toward oppressive social discourses, was another important factor associated with both measures of well-being among women post-treatment. While no studies to date have examined mental freedom in the cancer population, post-structural feminists, including Bronwyn Davies [39] and Valerie Walkerdine [40], have discussed how “gendered subjectivities are not shaped passively, but rather are actively developed as individuals personally take up discourses, discourses that shape their identity” [29]. A young woman with breast cancer, for example, may choose to resist and renegotiate normative performances of gender and beauty ideals (i.e., refusing reconstructive surgery). In Trachtenberg and Piran’s [41] qualitative study, which utilized a subset of participants from the current study, a 43-year-old woman with Stage II cancer, 13 months post-medical treatment, chose to resist and renegotiate her normative performances of femininity. In her interview, she stated, “I guess now that I have one breast it feels it’s so ludicrous that society is going to define beauty and I resent that … I am experimenting with this notion of: is beauty just totally socially constructed? In other words, can a scar where a breast was actually be perceived as a thing of beauty?” (p. 83). This pattern of results is not unique to women with breast cancer. Research of healthy women similarly indicated the disruptive effects of gender socialization on body and self-image, depression [42], embodiment [29], and disordered eating [43,44]. However, in light of the challenges encountered by young women who are diagnosed with breast cancer, it is important to consider the added toll of contending with the multiplicity of social expectations associated with femininity. For example, a strong focus on maintaining appearance standards that approximate idealized, objectified, and sexualized images of women in terms of weight, hair, and breasts can become oppressive and discouraging [45]. In a similar vein, idealized constructions of women as selfless caretakers, functioning perfectly at work and home, can stand in the way of much-needed self-care and disrupt self-esteem [15]. Further, the qualities of power and agency, attuned self-care, and the pursuit of one’s own passions, often discouraged among girls and women [23], could be important in asserting needs and choices related to treatment and healing. These processes are important to understand, as they have implications about providing health-promotion interventions with women treated for breast cancer.

From a clinical intervention standpoint, the results suggested the value of health care practitioners acknowledging and validating the psychosociocultural factors that impact women’s experiences in their bodies following breast cancer treatment(s). Health care practitioners who support women in strengthening their own critical stance towards gender-role expectations and related social discourses may have substantial benefit on their overall well-being. Interventions that target mental freedom by strengthening resistance to rigid gender roles have shown significant improvements in well-being among women with cancer [46,47]. Restoring Body Image after Cancer (ReBIC) [12] and its online version (i-ReBIC) [46] are manual-based group therapy interventions including a psychoeducational component that acknowledges, validates, and challenges present psychosocialcultural factors that impact women’s experiences following cancer treatments. Therapists leading the group intervention promote discussions about mental freedom, providing insight and strengthening their resistance to rigid gender roles. A randomized control trial conducted on ReBIC showed that women in the intervention group reported significantly less distress over their body appearance (*p* < 0.01), decreased body stigma (*p* < 0.01), and lower levels of breast-cancer-related concerns (*p* < 0.01) compared to women in the control group. This intervention further highlighted the value of utilizing women’s mental freedom to improve overall well-being following completion of cancer treatment(s).

A few notable limitations of the study should be addressed. The study included participants up to the age of 49 during the time of diagnosis. While this age range may be comparable to that of other studies that focus on younger samples of women with breast cancer, it was a broad range. There may be important differences within this age range in women’s well-being and the factors that shape it. While participants’ current age was used as a covariate in the regression analyses and was thus controlled, group-based analysis of the results may have marked differences related to varied age groups. Future studies could compare different age groups within a broader range (e.g., women aged 20–29 versus women aged 30–39). The study was also cross-sectional in design, limiting conclusions to be drawn on causality. Future studies could create a prospective study design to assess women’s experiences of gender socialization throughout varied stages of their cancer trajectory (i.e., before and after treatment completion). Future studies could also include qualitative studies to more comprehensively understand women’s internalized experiences of gender socialization across the cancer trajectory. Trachtenberg and Piran [5] conducted a separate qualitative study with semi-structured interviews to further understand women’s experiences of gender socialization across different stages of their cancer trajectory.

Furthermore, in order to maximize sample size, participants from all four stages of breast cancer were included. While the majority of participants were between Stage I-III (88.6%), a small subset of participants had a Stage IV (11.4%) diagnosis. Thus, these results cannot be generalized to individuals diagnosed with carcinoma in-situ or individuals with metastatic disease. The majority of participants were of European origin (62.3%) and generalizations regarding women of diverse social locations should be considered cautiously. The study did not consider the experiences of individuals who identify as non-binary. Future studies could explore gender socialization and well-being specifically among those who identify as non-binary.

## 5. Conclusions

The present study represented a novel exploration of four components of gender socialization used to assess young women’s well-being following treatment for breast cancer. Findings indicated that women who resisted the internalization of traditional gender-role beliefs, resisted self-objectification through body shaming and body surveillance, and endorsed a critical stance toward oppressive social discourses showed greater well-being following treatment. More studies are needed to examine the impact of gender socialization on the well-being of young people who are being diagnosed with and treated for breast cancer.

## Figures and Tables

**Table 1:** Summary of participant demographic and medical characteristics.

Demographic Information		Mean (SD)	Range	*N (%)*
Age		36.25 (5.89)	22–49	
Ethnicity	European origin			71 (62.8)
	African origin			4 (3.5)
	Asian origin			15 (13.3)
	Latin American or Hispanic origin			7 (6.2)
	None of the above			16 (14.2)
Living arrangements	Living with partner			79 (69.9)
	Living alone			19 (16.8)
	Separated, divorced, widowed			6 (5.2)
	None of the above			9 (8)
Highest level of education	Part of/completed high school			14 (12.4)
	Part of/completed university/college			64 (56.7)
	Graduate school			35 (31)
Current occupation status	Employed full-time			63 (55.8)
	Employed part-time			14 (12.4)
	Unemployed/on disability			12 (10.6)
	Self-employed			13 (11.5)
	Retired/homemaker			11 (9.8)
Medical Information				
Age at diagnosis	20–29			17 (15)
	30–39			56 (49.6)
	40–49			40 (35.4)
Months since treatment completed	1–12			65 (57.5)
	12–24			25 (22.1)
	25+			23 (20.4)
Stage of breast cancer	Stage 0–I			35 (31)
	Stage II			35 (31)
	Stage III–IV			43 (6.2)
Surgery type	Lumpectomy			41 (36.3)
	Mastectomy			64 (56.6)
	Other surgery			8 (7.1)
Reconstruction	Completed			30 (26.5)
	Not completed			75 (66.3)

**Table 2:** Means, standard deviations, and Cronbach alphas of study variables (*N* = 113).

	Mean (SD)	Possible Range	Cronbach Alpha
GRSS	99.88 (28.96)	30.00–163.00	0.86
OBCS		8.00–56.00	
Body shame	30.18 (15.01)		0.84
Body surveillance	32.14 (14.07)		0.81
MFS	116.10 (17.16)	61.00–149.00	0.90
SSS		10.00–40.00	
Care as Self-Sacrifice	23.67 (5.73)		0.89
Silencing the Self	21.87 (7.83)		0.73
FACT-B	137.45 (21.25)	86.78–179.78	0.91
EES	121.24 (18.25)	81.00–162.00	0.90

GRSS, Gender Role Socialization Scale; OBCS, Objectified Body Consciousness Scale; MFS, Mental Freedom Scale; SSS, Silencing the Self Scale; FACT-B, Functional Assessment of Cancer Therapy—Breast; EES, Experience of Embodiment Scale.

**Table 3:** Correlations among well-being and gender socialization scores.

Gender Socialization Measures	FACT-B	EES
GRSS	−0.45 **	−0.59 **
OBCS—Body Shame	−0.32 **	−0.63 **
OBCS—Body Surveillance	−0.23 *	−0.54 **
MFS	0.42 **	0.57 **
SSS—Care as Self-Sacrifice	−0.20 *	−0.31 **
SSS—Silencing the Self	−0.22 *	−0.29 **

GRSS, Gender Role Socialization Scale; OBCS, Objectified Body Consciousness Scale; MFS, Mental Freedom Scale; SSS, Silencing the Self Scale; FACT-B, Functional Assessment of Cancer Therapy—Breast; EES, Experience of Embodiment Scale. ** *p <* 0.01 level (2-tailed), * *p <* 0.05 level (2-tailed).

**Table 4:** Hierarchical regression analysis for gender socialization variables predicting FACT-B scores.

Variable	*B*	*SE_B_*	*Β*	*T*	*R* ^2^	Δ*R*^2^	Δ*F*	*df*
1. Covariates					0.404	0.404	3.960 **	5, 107
Age	0.46	0.30	0.15	1.54				
FT	13.67	5.16	0.32	2.65 **				
PT/SE	15.38	6.20	0.31	2.48 *				
Stage 0–I	10.79	4.53	0.24	2.38 *				
Stage II	0.08	4.54	0.00	0.02				
2. Main Effects					0.637	0.233	6.826 ***	6, 101
GRSS	−0.28	0.09	−0.39	−3.04 **				
OBCS-Surv	−1.37	1.76	−0.75	−0.78				
OBCS-BS	1.37	1.89	0.09	0.73				
MFS	0.31	0.14	0.24	2.27 *				
SSS-1	0.45	0.40	0.12	1.10				
SSS-2	−0.12	0.27	−0.04	−0.43				

Note. Age = current age; FT = full-time employed; PT/SE = part-time/self-employed; Stage 0-I= Stage 0 and I cancer; Stage II = Stage II-IV cancer; GRSS = Gender Role Socialization Scale; OBCS-Surv = Objectified Body Consciousness Scale—Body Surveillance; OBCS-BS = Objectified Body Consciousness Scale—Body Shame; MFS = Mental Freedom; SSS-1= Silencing the Self—Care as Self-Sacrifice Subscale; SSS-2= Silencing the Self –Silencing the Self Subscale. * *p* < 0.05. ** *p* < 0.01. *** *p* < 0.001.

**Table 5:** Hierarchical regression analysis for gender socialization variables predicting EES scores.

Variable	*B*	*SE_B_*	*β*	*t*	*R* ^2^	Δ*R*^2^	Δ*F*	*df*
1. Covariates					0.333	0.333		5, 107
Age	0.33	0.26	0.12	1.25			2.667 **	
FT	7.37	4.58	0.20	1.61				
PT/SE	13.08	5.52	0.30	2.37 *				
Stage 0-I	6.21	4.04	0.16	1.54				
Stage II	−0.49	4.06	−0.01	−0.12				
2. Main Effects					0.800	0.467	22.53 ***	6, 101
GRSS	−0.10	0.06	−0.16	−1.66				
OBCS—Surv	−4.81	1.19	−0.30	−4.03 ***				
OBCS—BS	−3.50	1.29	−0.25	−2.71 **				
MFS	0.24	0.09	0.21	2.65 **				
SSS—1	0.00	0.27	0.00	−0.00				
SSS—2	−0.08	0.18	−0.03	−0.43				

Note. Age = current age; FT = full-time employed; PT/SE = part-time/self-employed; Stage 0-I= Stage 0 and I cancer; Stage II = Stage II-IV cancer; GRSS = Gender Role Socialization Scale; OBCS-Surv = Objectified Body Consciousness Scale—Body Surveillance; OBCS-BS = Objectified Body Consciousness Scale—Body Shame; MFS = Mental Freedom; SSS-1= Silencing the Self—Care as Self-Sacrifice Subscale; SSS-2= Silencing the Self –Silencing the Self Subscale. * *p* < 0.05, ** *p* < 0.01, *** *p* < 0.001.

## Data Availability

The data presented in this study are available on request from the corresponding author.
